# Casein kinase 2 α and β subunits inversely modulate ABA signal output in *Arabidopsis* protoplasts

**DOI:** 10.1007/s00425-018-2919-5

**Published:** 2018-05-24

**Authors:** Yukari Nagatoshi, Miki Fujita, Yasunari Fujita

**Affiliations:** 1 Biological Resources and Post-harvest Division, Japan International Research Center for Agricultural Sciences (JIRCAS), Tsukuba, Ibaraki 305-8686 Japan; 2 RIKEN Center for Sustainable Resource Science, Tsukuba, Ibaraki 305-0074 Japan; 3 Graduate School of Life and Environmental Sciences, University of Tsukuba, Tsukuba, Ibaraki 305-8572 Japan

**Keywords:** ABA-responsive-elements, Abscisic acid signaling, *Arabidopsis thaliana*, AREB/ABFs, Protoplast transient expression system, SnRK2

## Abstract

Our transient gene expression analyses in *Arabidopsis* protoplasts support the view that CK2αs and CK2βs positively and negatively modulate ABRE-dependent gene expression, respectively.

The phytohormone abscisic acid (ABA) regulates the expression of thousands of genes via ABA-responsive elements (ABREs), and has a crucial role in abiotic stress response. Casein kinase II (CK2), a conserved Ser/Thr protein kinase in eukaryotes, is essential for plant viability. Although the CK2 has been known as a tetrameric holoenzyme comprised of two catalytic α and two regulatory β subunits, each of the two types of subunits has been proposed to have independent functions. The *Arabidopsis* genome encodes four α subunits (*CK2α1*, *CK2α2*, *CK2α3*, *CK2α4*) and four β subunits (*CK2β1*, *CK2β2*, *CK2β3*, *CK2β4*). There is a growing body of evidence linking CK2 to ABA signaling and abiotic stress responses. However, the roles of each CK2 subunit in ABA signaling remain largely elusive. Using the transient expression system with the core ABA signaling components in *Arabidopsis* leaf mesophyll protoplasts, we show here that CK2α1 and CK2α2 (CK2α1/2) positively modulate ABRE-dependent gene expression as ABA signal output in ABA signaling, whereas all four CK2βs negatively modulate the ABRE-dependent gene expression mediated by subclass III SnRK2–AREB/ABF pathway and by CK2α1/2. These data indicate that CK2α1/2 and CK2βs positively and negatively modulate ABA signal output, respectively, suggesting that the quantitative balance of CK2 subunits determines the ABA signal output in plants. Given that CK2s act as pleiotropic enzymes involved in multiple developmental and stress–responsive processes, our findings suggest that CK2 subunits may be involved in integration and coordination of ABA-dependent and -independent signaling.

**Electronic supplementary material:**

The online version of this article (10.1007/s00425-018-2919-5) contains supplementary material, which is available to authorized users.

## Introduction

The phytohormone abscisic acid (ABA) has crucial roles in a broad range of plant developmental processes and environmental stress responses (Cutler et al. 2010; Raghavendra et al. 2010; Miyakawa et al. 2013; Yoshida et al. 2015). Cellular dehydration during seed maturation and post-germination growth enhances endogenous ABA levels, which modulate the expression of many dehydration-responsive genes (Fujita et al. 2011). Approximately 10% of protein-coding genes in *Arabidopsis thaliana* plants are modulated by ABA, which is a much larger gene subset than that modulated by other phytohormones (Nemhauser et al. 2006). Many ABA-responsive genes carry conserved G-box-like *cis*-acting ABA-responsive elements (ABREs, PyACGTGG/TC) in their promoter regions (Mundy et al. 1990; Busk and Pages 1998; Hattori et al. 2002; Zhang et al. 2005; Gomez-Porras et al. 2007). ABA binds to the pyrabactin resistance1/PYR1-like/regulatory components of ABA receptor (PYR/PYL/RCAR) proteins to form ternary complexes with group-A protein phosphatase 2Cs (PP2Cs) (Ma et al. 2009; Miyazono et al. 2009; Nishimura et al. 2009; Park et al. 2009; Santiago et al. 2009). In the presence of ABA, the subclass III sucrose nonfermenting 1 related protein kinase 2 (SnRK2) is released from the PP2C-mediated negative regulation and phosphorylates basic leucine zipper (bZIP) transcription factors such as ABRE-binding protein/ABRE-binding factors (AREB/ABFs), which then activate the expression of ABA-responsive genes. Taken together with the recent results from a systemic study of a transcriptional network in response to ABA (Song et al. 2016), these findings indicate that ABRE-dependent gene expression constitutes the major ABA-responsive gene expression as ABA signal output in response to dehydration stress.

Casein kinase II (CK2) is a Ser/Thr protein kinase that is evolutionarily conserved in all eukaryotes. CK2 is essential for cell proliferation and survival, and is emerging as a potential target for anticancer pharmaceuticals (Mulekar and Huq 2014). CK2 is known to exist as a tetrameric holoenzyme composed of two catalytic α and two regulatory β subunits (Litchfield. 2003). On the other hand, several lines of evidence suggest that each of two types of subunit can exist independently and have independent functions as a monomer (Filhol et al. 2004). The *Arabidopsis* genome contains four α subunits (*CK2α1*, *CK2α2*, *CK2α3*, *CK2α4*) and four β subunits (*CK2β1*, *CK2β2*, *CK2β3*, *CK2β4*) genes. Recent molecular genetics and biochemical analyses suggest that CK2 is involved in ABA signaling and abiotic stress responses (for review: Vilela et al. 2015b). So far, studies of CK2 knockout mutants and RNAi lines suggest that CK2αs and CK2β1 are positive regulators of ABA responses (Mulekar et al. 2012; Mulekar and Huq 2014; Wang et al. 2014; Yuan et al. 2017), whereas biochemical studies suggest that CK2 negatively regulates SnRK2-mediated ABA signaling (Vilela et al. 2015a). It is thus controversial whether casein kinase II subunits (CK2s) positively or negatively regulate ABA signaling in plants. This discrepancy may be due to a lack of appropriate multiple knockout mutants of CK2α and CK2β required to determine the precise roles in ABA signaling, though CK2s have redundant functions as pleiotropic regulators of cell cycle, light signaling, circadian rhythms, flowering time, and hormone responses (Mulekar and Huq 2014). In fact, there are the adjacent positions of *CK2α3* (At2g23080) and *CK2α4* (At2g23070) on the chromosome, and *ck2β2* and *ck2β3* T-DNA insertion lines are not available. It is, therefore, challenging to determine the functions and roles of CK2s only by a genetic approach. In this study, to help overcome this problem, we used *Arabidopsis* leaf mesophyll protoplasts as a transient expression system to examine whether CK2s affect ABRE-dependent gene expression as an ABA signal output in ABA signaling. Here, we report that CK2αs positively modulate ABRE-dependent gene expression dependently and independently of the core ABA signaling pathway in the presence and the absence of exogenous ABA, respectively, whereas CK2βs negatively modulate ABRE-dependent gene expression in an exogenous ABA-independent manner.

## Materials and methods

### Plant materials and growth conditions

For transient expression analyses, *A. thaliana* L. accession Columbia-0 (Col-0, CS60000) and two triple knockout mutant lines *areb1/2abf3* (Yoshida et al. 2010) and *srk2d/e/i* (Fujita et al. 2009; Nakashima et al. 2009) were grown in soil in pots (9.5 cm diameter) in an environmentally controlled chamber (CF-405S, TOMY, Osaka, Japan) at 22 °C under a 12-h light/12-h dark cycle (70 ± 20 μmol photons m^−2^ s^−1^). For transient expression analysis (cf. Fig. 2c), CS60000 were grown on GM agar plates to maintain high humidity as described previously (Fujita et al. 2005) with a 16-h light/8-h dark cycle (40 ± 10 µmol photons m^−2^ s^−1^). Similar results were obtained with these two methods.

### Isolation of *Arabidopsis* leaf mesophyll protoplasts

*Arabidopsis* leaf mesophyll protoplasts were isolated as described previously (Yoo et al. 2007; Wu et al. 2009) with minor modifications. The leaves were collected from 2.5- to 4-week-old *Arabidopsis* plants. The upper epidermal leaf surfaces were affixed onto 19-mm width vinyl tape NO200-19-24 (Yamato, Tokyo, Japan). The basal epidermal leaf surfaces were affixed onto the same tape. The two strips of tape were then carefully torn away from each other to remove the lower epidermal cell layer. The peeled leaves attached to the tape were immersed in a 5 mL tube containing 4 mL of enzyme solution [20 mM Mes, pH 5.7, 1.5% (w/v) cellulase R10, 0.4% (w/v) macerozyme R10, 0.4 M mannitol and 20 mM KCl, and 10 mM CaCl_2_, 0.1% BSA]. Three tape strips (approximately 30 mm length per strip) were immersed in one tube. The tubes were gently shaken RT-50 (Taitec, Koshigaya, Japan) for 20–60 min. After the protoplasts were released into the solution, the tapes were removed from the solution and the protoplasts were collected by centrifuging at 100*g* for 2 min.

### Transient expression analysis

**Fig. 1:**
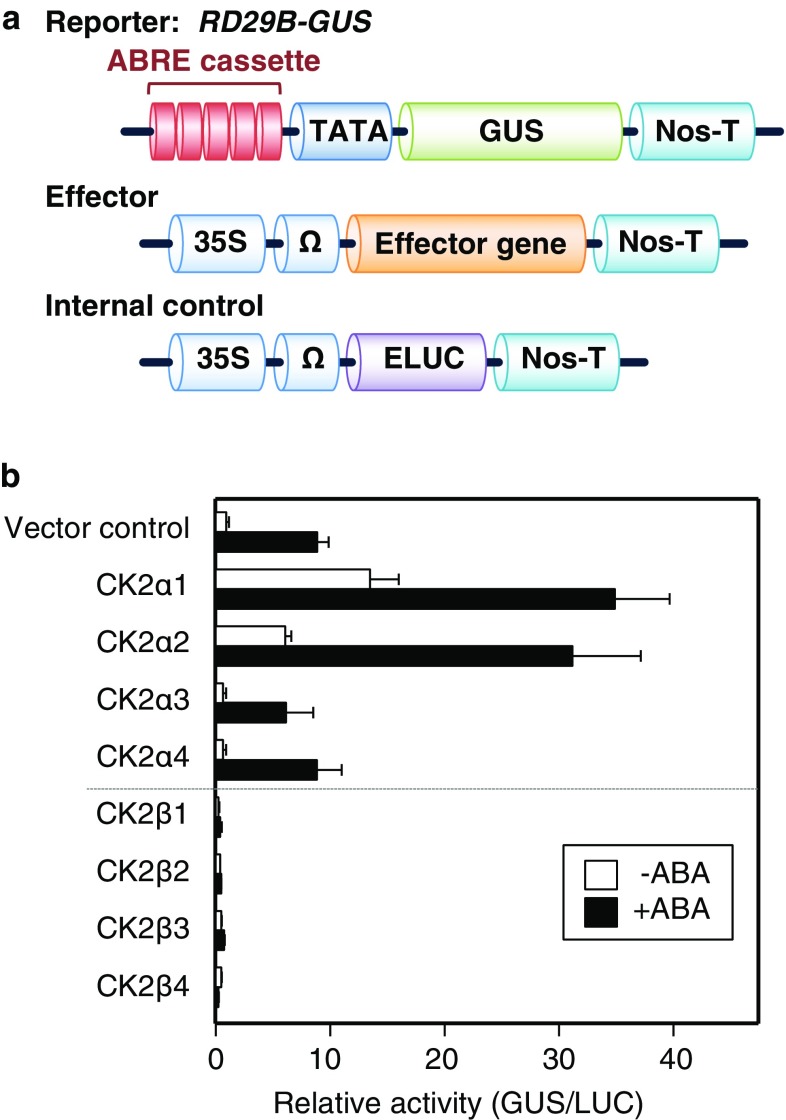
CK2α1/2 and CK2βs positively and negatively modulate ABRE-dependent gene expression in *Arabidopsis* leaf mesophyll protoplasts, respectively. **a** Scheme of reporter, effector, and internal control constructs used for transient expression analysis. The *RD29B*-*GUS* reporter construct carries five tandem copies of a 77-bp *Arabidopsis RD29B* promoter fragment containing two ABRE motifs fused to the *GUS* gene. The effector constructs contain the Cauliflower mosaic virus (CaMV) 35S promoter and tobacco mosaic virus Ω sequence fused to each cDNA fragment of interest. The *pBI35SΩ*-*ELUC* reporter construct was co-transfected as an internal control for transfection efficiency. **b** CK2α1/2 and CK2βs have inverse roles in ABRE-dependent gene expression in *Arabidopsis* leaf mesophyll protoplasts. Protoplasts were isolated from WT leaves. ‘Relative activity’ indicates combined expression relative to the value obtained from the vector control. Open bars or filled bars indicate without ABA or with 2.0 μM ABA, respectively. Error bars indicate SD (*n* = 4). Experiments were performed at least three times, and a representative result is shown

**Fig. 2:**
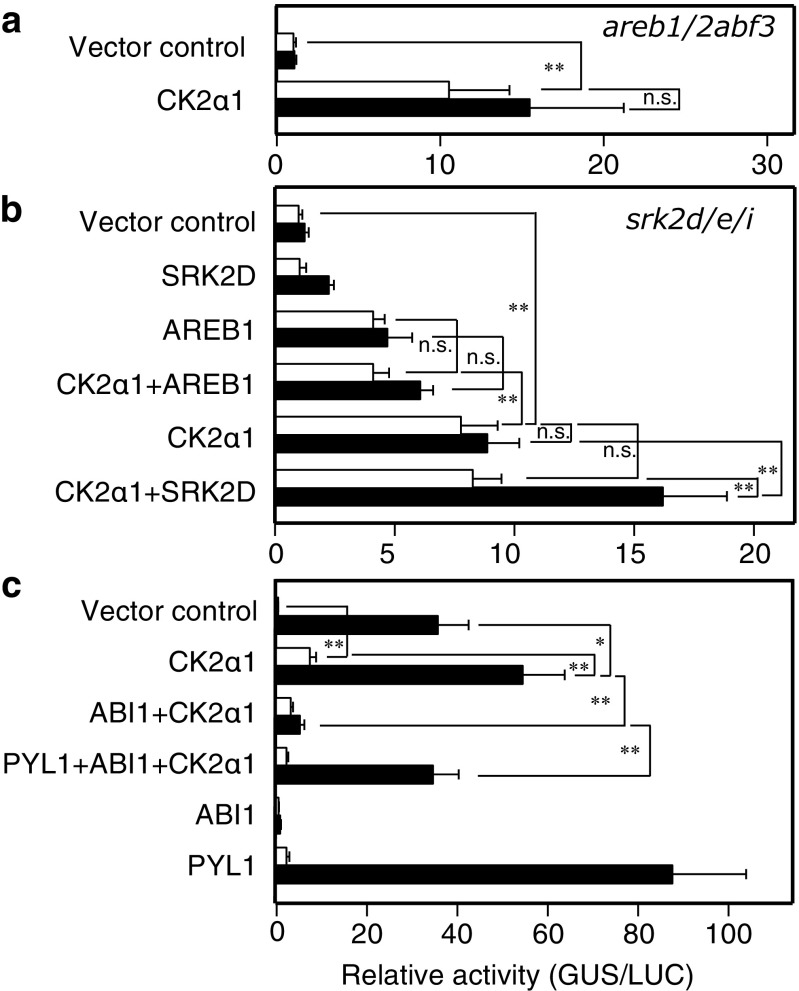
CK2α1 positively modulates ABRE-dependent gene expression independently and dependently of the core ABA signaling pathway in the absence and presence exogenous of ABA, respectively. **a** CK2α1 positively modulates ABRE-dependent gene expression in an AREB1/2ABF3-independent and -dependent manner in the absence and presence of exogenous ABA, respectively. Protoplasts were isolated from *areb1/2abf3* triple mutant leaves. **b** CK2α1 positively modulates ABRE-dependent gene expression in a SnRK2D/E/I-independent and -dependent manner in the absence and presence of exogenous ABA, respectively. Protoplasts were isolated from *srk2d/e/i* triple mutant leaves. **c** PYL1 and ABI1 affect the stimulatory effect of CK2α1 on ABRE-dependent gene expression in the presence of exogenous ABA. Protoplasts were isolated from WT leaves. Open bars or filled bars indicate without ABA or with 2.0 μM ABA, respectively. Error bars indicate SD (*n* = 4). Experiments were performed at least three times, and a representative result is shown. *P < 0.05, **P < 0.01, *n.s.* no significant difference

**Fig. 3:**
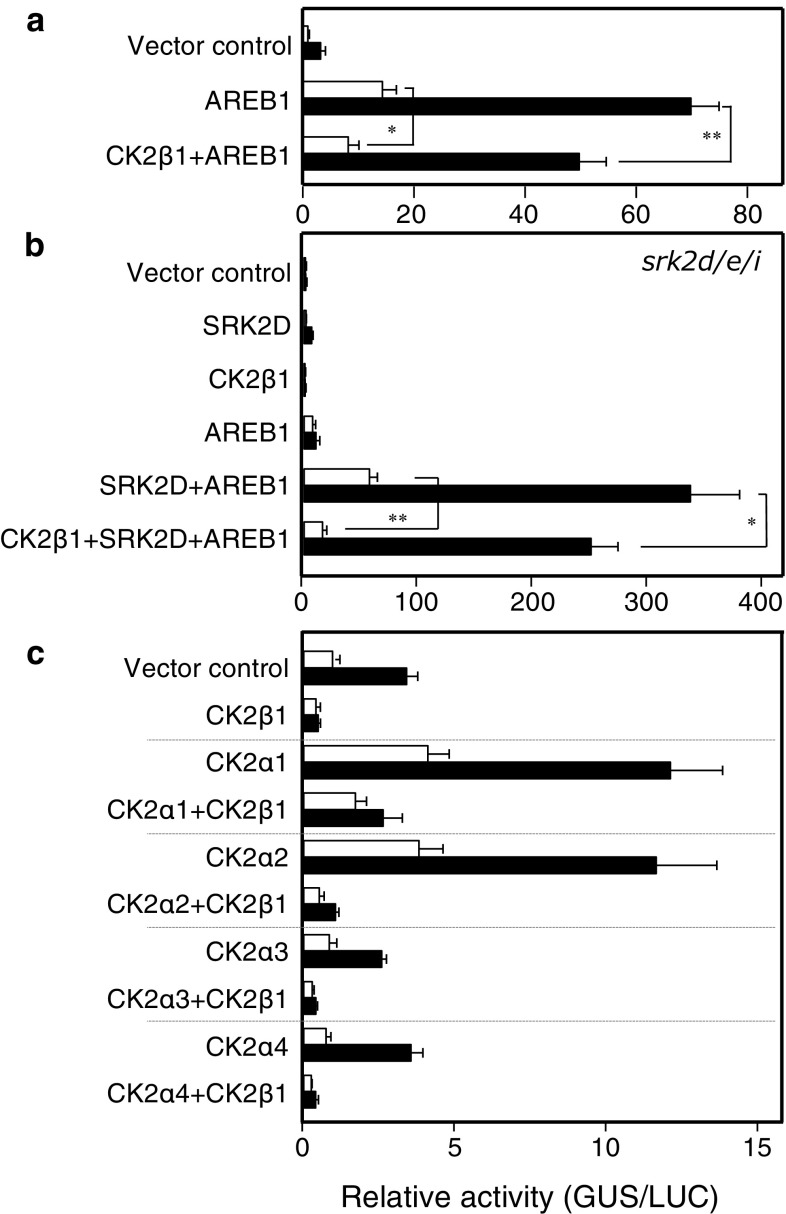
CK2β1 negatively modulates ABRE-dependent gene expression mediated by AREB–SnRK2 pathway and by CK2αs in an ABA-independent manner. **a** CK2β1 negatively modulates AREB1-mediated ABRE-dependent gene expression. Protoplasts were isolated from WT leaves. **b** CK2β1 negatively modulates AREB/SnRK2-mediated ABRE-dependent gene expression. Protoplasts were isolated from *srk2d/e/i* triple mutant leaves. **c** CK2β1 negatively modulates CK2α-mediated ABRE-dependent gene expression. Protoplasts were isolated from WT. Open bars or filled bars indicate without ABA or with 2.0 μM ABA, respectively. Error bars indicate SD (*n* = 4). Experiments were performed at least three times, and a representative result is shown. *P < 0.05, **P < 0.01, *n.s.* no significant difference

Transient expression analysis was performed using protoplasts derived from *Arabidopsis* leaf mesophyll cells as described previously (Yoo et al. 2007) with minor modifications. Plasmid DNA was prepared using a plasmid DNA purification kit (Qiagen). The β-glucuronidase (GUS) reporter plasmid, *RD29B*-*GUS* (Uno et al. 2000), was co-transfected with effector plasmids and with the *pBI35SΩ*-*ELUC* (Mizoi et al. 2013) plasmid as an internal control to normalize protoplast transfection efficiency. The *pBI35S*-*AREB1* plasmid (Fujita et al. 2005) was used as an effector plasmid. For analysis, *RD29B*-*GUS* (5.0 μg of plasmid per transfection) and *pBI35SΩ*-*ELUC* (1.0 μg per transfection) were used. Each transfection used 2.5 μg of effector plasmid, except for ABI1 (1.5 μg), AREB1 (1.0 μg) and CK2β (1.0 μg) in Fig. 3a, and SRK2D (0.625 μg) in Fig. 3b, per transfection. Total amounts of effector DNA per experiment, which include effector plasmids alone or combined with the vector control plasmid *pSKX* for transient expression analysis, were as follows: 2.5 μg (Fig. 1b), 5.0 μg (Figs. 2a, 3a, c), 6.5 μg (Fig. 2c), 7.5 μg (Fig. 2b), and 5.625 μg (Fig. 3b). ‘Relative activity’ indicates combined expression relative to the value obtained from the vector control. After transfection, protoplasts were incubated in 2 mL tubes at 22 °C for 14–18 h under dark conditions without ABA or with 2.0 μM ABA.

### Plasmid construction for transient expression analysis

The primers used for DNA fragment amplification are listed in Supplementary Table S1.

#### *pSKX*

*pSKX*-based vectors were used for transient expression analysis of selected effectors, except for *pBI35S*-*AREB1*. A DNA fragment including *El2*-*35SΩ*-*NosT* was amplified by PCR from *pGKX* (Qin et al. 2008) using the forward and reverse *Pst*I–*Hin*dIII linker primer pair (pGKXEl2-5′PstHind and pGrMCS-3′). The resulting PCR fragment was digested with *Pst*I, subjected to end blunting and digested with *Kpn*I, and inserted into the end-blunted *Sac*I and *Kpn*I site of *pBluescriptIISK(*−*)* to produce *pSK*-*EI2*-*35SΩ* (named *pSKX*).

#### *pSKX*-*CK2α*s and *pSKX*-*CK2β*s

For *pSKX*-*CK2α1*, *pSKX*-*CK2α2*, *pSKX*-*CK2α3*, *pSKX*-*CK2α4*, *pSKX*-*CK2β1*, *pSKX*-*CK2β2*, *pSKX*-*CK2β3*, and *pSKX*-*CK2β4*, DNA fragments of full-length open reading frames (FL ORFs) of *CK2α*s and *CK2β*s were PCR-amplified from their cDNA with *Bam*HI–*Sac*I linker primers. The resulting PCR products were digested with *Bam*HI and *Sac*I, and cloned into the *Bam*HI and *Sac*I sites of the plasmid vector *pSKX*.

#### *pSKX-ABI1dMyc*

The *ABI1* FL ORF was PCR-amplified from its cDNA using ABI1F-*Not*I and ABI1R1-*Stu*I primers, and the fragment was purified by ethanol precipitation. The resulting fragment was PCR-amplified using ABI1F-*Not*I and ABI1R4-dMyc-*Sma*I primers, purified, and further amplified using ABI1F-*Not*I and ABI1R5-dMyc-*Sma*I primers. The resulting PCR product was digested with *Not*I and *Sma*I, and cloned into the *Not*I and *Eco*RV sites of *pSKX* to produce *pSKX*-*ABI1dMyc*.

#### *pSKX-SRK2DFlag*

The FL ORF DNA of SRK2D was PCR-amplified from its cDNA using SRK2DF-*Not*Ia and SRK2DR1-*Stu*Ia primers. The resulting fragment was purified by ethanol precipitation, and then subjected to further PCR amplification using SRK2DF-*Not*Ia and SRK2DR2-Flag-*Eco*RV primers. The resulting PCR product was then digested with *Not*I and *Eco*RV, and cloned into the *Not*I and *Eco*RV sites of *pSKX* to produce *pSKX*-*SRK2DFlag*.

#### *pSKX-HisPYL1*

The FL ORF DNA of *PYL1* was PCR-amplified from its cDNA using His-*Nco*I-PYL1F1a and PYL1R1-*Eco*RVa primers. The resulting fragment was purified by ethanol precipitation, and then subjected to further PCR amplification using *Not*I-His-*Nco*I-PYL1F2 and PYL1R1-*Eco*RVa primers. The resulting PCR product was digested with *Not*I and *Eco*RV, and cloned into the *Not*I and *Eco*RV sites of *pSKX* to produce *pSKX*-*HisPYL1*.

### Phylogenetic analysis

Amino acid sequences were aligned and clustered using Clustal X2.1 (http://www.genome.jp/tools-bin/clustalw) (Larkin et al. 2007). The phylogenetic tree was constructed using MEGA7 (Kumar et al. 2016).

## Results

### CK2αs and CK2βs inversely modulate ABRE-dependent gene expression

ABRE-dependent gene expression plays a pivotal role in ABA-responsive gene expression as ABA signal output in response to dehydration stress (Fujita et al. 2011). Although there is a growing body of evidence linking CK2 to ABA signaling and abiotic stress responses (Vilela et al. 2015b), it remains unclear how CK2αs and CK2βs are involved in ABRE-dependent gene expression in ABA signaling. To examine the roles of all eight CK2s in ABA-responsive ABRE-dependent gene expression, we performed transient expression assays in *Arabidopsis* leaf mesophyll protoplasts using a β-glucuronidase (GUS) reporter gene, *RD29B*-*GUS*, driven by ABRE *cis*-elements derived from the ABA-responsive *RD29B* promoter (Uno et al. 2000) (Fig. 1a). Transfection of CK2α1 or CK2α2 induced *RD29B*-*GUS* expression in leaf mesophyll protoplasts in the presence and the absence of exogenous ABA compared with the vector control, whereas transfection of CK2α3 or CK2α4 did not (Fig. 1b). The findings are consistent with the observation that CK2α1 and CK2α2 are the closest homologs in the phylogenetic tree (Fig. S1), and only they have putative cleavage sites in the N-terminal region (Fig. S2). These collective data support the view that CK2α1 and CK2α2 (CK2α1/2) positively modulates ABRE-dependent gene expression (Fig. 1b). By contrast, CK2β transfection suppressed *RD29B*-*GUS* expression in leaf mesophyll protoplasts compared with the vector control in the presence and the absence of exogenous ABA, indicating that all four CK2βs negatively modulates ABRE-dependent gene expression in an ABA-independent manner (Fig. 1b). Collectively, these data indicate that CK2α1/2 and CK2βs positively and negatively modulate ABRE-dependent gene expression, respectively.

### CK2α1 positively modulates ABRE-dependent gene expression

The core ABA signaling components (PYR/PYL/RCARs, group-A PP2Cs, subclass III SnRK2s, and AREB/ABF transcription factors) are necessary and sufficient for ABA perception, signaling, and ABA-responsive ABRE-dependent gene expression in a transient expression system based on *Arabidopsis* leaf mesophyll protoplasts (Fujii et al. 2009). To investigate how the CK2α1/2 positively modulates ABRE-dependent gene expression, we evaluated the effects of CK2α1 on *RD29B*-*GUS* expression in protoplasts derived from three different genotypes: an AREB/ABF subfamily triple mutant *areb1/2abf3* (Yoshida et al. 2010), a subclass III SnRK2 subfamily triple mutant *srk2d/e/i* (Fujita et al. 2009; Nakashima et al. 2009), or wild-type (WT) *Arabidopsis*. Significant ABA-dependent induction of *RD29B*-*GUS* expression caused by transfection of CK2α1 could not be observed in *areb1/2abf3* or *srk2d/e/i* protoplasts (Fig. 2a, b) unlike the transfection of CK2α1 in WT protoplasts (Figs. 1b, 2c), whereas co-transfection of SRK2D/SnRK2.2 (SRK2D) with CK2α1 recovered the ABA-dependent induction of *RD29B*-*GUS* expression in *srk2d/e/i* protoplasts (Fig. 2b). These data suggest that CK2α1 positively modulates ABRE-dependent gene expression in an AREB/SnRK2-dependent manner in the presence of ABA.

By contrast, even in the absence of exogenous ABA, transfection of CK2α1 alone induced significant level of *RD29B*-*GUS* expression in either *areb1/2abf3*, *srk2d/e/i*, or WT protoplasts compared with the vector control in each experiment (Figs. 1b, 2) and the co-transfection of CK2α1 with SRK2D did not affect *RD29B*-*GUS* expression in *srk2d/e/i* protoplasts compared with the transfection of CK2α1 alone in the *srk2d/e/i* protoplasts (Fig. 2b). These data support the notion that CK2α1 positively modulates ABRE-dependent gene expression in an AREB/SnRK2-independent manner in the absence of exogenous ABA. Indeed, in the absence of exogenous ABA in *srk2d/e/i* protoplasts, co-transfection of AREB1 with CK2α1 did not affect *RD29B*-*GUS* expression compared with the transfection of AREB1 alone (Fig. 2b), indicating that CK2α1 instead of SRK2D does not directly activate ABRE-dependent gene expression via AREB-mediated pathway in the absence of exogenous ABA. Moreover, in the absence of exogenous ABA in *srk2d/e/i* protoplasts, co-transfection of CK2α1 with AREB1 down-regulated *RD29B*-*GUS* expression compared with the transfection of CK2α1 alone (Fig. 2b), showing that the overexpression of AREB1 counteracts ABRE-dependent gene expression induced by CK2α1 in the absence of exogenous ABA. These data suggest that ABRE-binding transcription factors other than AREB/ABFs used in this study, which can compete with ABRE-dependent gene expression by AREB1, may be involved in CK2α1-mediated ABRE-dependent gene expression in the absence of exogenous ABA.

Next, we investigated the effects of CK2α1 combined with group-A PP2C and the PYR/PYL/RCAR ABA receptor on the induction of ABA-dependent induction of *RD29B*-*GUS* expression in WT protoplasts. As reported previously (Fujii et al. 2009), co-transfection of AREB1 with ABI1 inhibited *RD29B*-*GUS* expression in the presence of ABA, whereas co-transfection of PYL1 together with AREB1 and ABI1 partially recovered ABA-dependent induction of *RD29B*-*GUS* expression (Fig. S3). Co-transfection of CK2α1 with ABI1 inhibited *RD29B*-*GUS* expression, while co-transfection of CK2α1 together with ABI1 and PYL1 partially recovered ABA-dependent induction of *RD29B*-*GUS* expression (Fig. 2c). These data indicated that PYL1 and ABI1 affect the stimulatory effect of CK2α1 on ABRE-dependent gene expression in the presence of exogenous ABA. Together, our data support the hypothesis that CK2α1 positively modulates ABRE-dependent gene expression dependently of the core ABA signaling pathway in the presence of ABA.

### CK2β1 negatively modulates ABRE-dependent gene expression mediated by AREB–SnRK2 pathway and by CK2αs

To elucidate the mechanism of CK2β-mediated suppression of ABRE-dependent gene expression, we evaluated the effects of CK2β1 co-transfection with AREB1 and/or SRK2D on ABA-mediated *RD29B*-*GUS* expression in *srk2d/e/i* and WT protoplasts. Co-transfection of CK2β1 with AREB1 suppressed *RD29B*-*GUS* expression that was induced by transfection of AREB1 alone with or without ABA treatment (Fig. 3a), indicating that CK2β1 negatively modulates AREB1-mediated ABRE-dependent gene expression. Co-transfection of CK2β1 with SRK2D and AREB1 attenuated *RD29B*-*GUS* expression that was induced by co-transfection of SRK2D and AREB1 in *srk2d/e/i* protoplasts with or without ABA treatment, indicating that the CK2β1 negatively modulates AREB/SnRK2-mediated ABRE-dependent gene expression (Fig. 3b). These results were consistent with a previous report that CK2 is involved in negative regulation of SnRK2 through enhancing SnRK2 interaction with PP2C and degradation (Vilela et al. 2015a). Finally, to determine whether CK2α and CK2β coordinately affect ABRE-dependent gene expression, we analyzed the effects of CK2β1 co-transfection with CK2αs on *RD29B*-*GUS* expression. Co-transfection of CK2β1 with CK2α1, CK2α2, CK2α3, or CK2α4 strongly suppressed *RD29B*-*GUS* expression compared with the transfection of CK2α1-4 alone with or without ABA treatment (Fig. 3c). These data indicated that CK2β1 negatively modulates CK2α-mediated ABRE-dependent gene expression.

## Discussion

Here, we show that CK2α1/2 positively modulates ABRE-dependent gene expression dependently and independently of the core ABA signaling pathway in the presence and absence of exogenous ABA in *Arabidopsis* protoplasts, respectively (Figs. 1, 2). By contrast, the CK2βs negatively modulate ABRE-dependent gene expression mediated by AREB–SnRK2 pathway and by CK2αs in an exogenous ABA-independent manner in *Arabidopsis* protoplasts (Figs. 1, 3). These findings indicate that the CK2 α and β subunits inversely modulate ABRE-dependent gene expression as ABA signal output in *Arabidopsis* protoplasts, suggesting that the quantitative balance of CK2 subunits determines the ABA signal output in plants. However, it remains unclear whether the observed effects result from CK2 monomer function independently of holoenzyme or the interference with CK2 tetramer assembly. Given that the model proposed by Vilela et al. (2015a) in which CK2 negatively regulates ABA signaling through promoting SnRK2 degradation and enhancing SnRK2 interaction with PP2C, our data suggest a stimulatory effect of the catalytic subunits CK2α1 and CK2α2 which may be antagonized by the regulatory subunit CK2β1. Further research is required to clarify the regulatory mechanism of CK2 subunits in ABA signaling.

Our results show that in both the presence and absence of exogenous ABA, CK2s function as modulators of ABRE-dependent gene expression in *Arabidopsis* protoplasts (Figs. 1, 2, 3). This is in accordance with the previous findings that CK2 functions as a housekeeping gene (Mulekar and Huq 2014; Vilela et al. 2015b). Although so far CK2 has been identified as a negative regulator of ABA-activated SnRK2s in the core ABA signaling pathway (Vilela et al. 2015a), the roles and functions of CK2 subunits in the absence of exogenous ABA were unclear. We show here that CK2 α and β subunits positively and negatively modulate ABRE-dependent gene expression in an AREB/SnRK2-independent manner in the absence of exogenous ABA, respectively (Figs. 1, 2, 3). Since in the absence of exogenous ABA, CK2α1 instead of SnRK2 does not directly activate ABRE-dependent gene expression through AREB-mediated pathway, and the overexpression of AREB1 counteracts ABRE-dependent gene expression induced by CK2α1 (Fig. 2b), our collective data support the hypothesis that ABRE-binding transcription factors other than AREB/ABFs used in this study may activate ABRE-dependent gene expression downstream of CK2α1 in the absence of exogenous ABA. In contrast, considering the negative regulation model of CK2 in ABA signaling (Vilela et al. 2015a, b), the results suggest that negative modulation of ABRE-dependent gene expression by CK2β may be involved in ABRE/SnRK2-mediated pathway through the negative regulation of SnRK2 in the absence of exogenous ABA.

Thus, our analyses also provide the evidence that the protoplast transient expression system based on the ABRE-dependent gene expression is a useful tool to help overcome the problem in the limited genetic tools. On the basis of our results, combination studies of limited CK2s mutants with techniques of RNA silencing or CRISPR/Cas9, and transient expression analyses using the other marker genes, would provide more supportive information. Considering that CK2 acts as a pleiotropic enzyme involved in multiple developmental and stress–responsive processes and also functions as a housekeeping kinase regulating protein turnover in ABA signaling, the data presented here support the view that CK2 is a key modulator of crosstalk between ABA signaling and the other signaling pathways implicated in several other processes such as cell cycle, light signaling, and circadian rhythms. Together, this study suggests that CK2 subunits are involved in synergistically coordinating ABA-dependent and -independent signaling to modulate ABA signal output. Further works on CK2 interactors are needed to map novel CK2-mediated signaling networks that fine-tune ABA signal output in plants.

### *Author contribution statement*

YN, MF, and YF designed the experiments and constructed the plasmids. YN performed the experiments and analyzed the data. YN and YF wrote the paper. YF conceived the research. All authors reviewed the manuscript.

## Electronic supplementary material

Below is the link to the electronic supplementary material.

Suppl. Table S1 Oligonucleotide primers used in this study. Suppl. Fig. S1 Phylogenetic tree of CK2αs and CK2βs in *Arabidopsis.* The neighbor-joining phylogenetic tree (Saitou and Nei 1987) was created using MEGA7 (Kumar et al. 2016). The optimal tree with the sum of branch length = 1.25324778 is shown. Bootstrap values (1000 replicates) are shown next to the branches (Felsenstein 1985). Evolutionary distances were computed using the p-distance method (Nei and Kumar 2000); units represent the number of amino acid differences per site. The subcellular localization of each CK2 is based on previous reports (Salinas et al. 2006; Perales et al. 2006; Portoles and Mas 2010: Mulekar and Huq 2015). CK2αs and CK2βs are shaded in pink and in yellow, respectively. Suppl. Fig. S2 Alignment of CK2 amino acid sequences in *Arabidopsis.* Comparison of amino acid sequences of CK2αs (a) and CK2βs (b). Conservation ratio at each site is shown by shading (black, 100%; gray, 75%). Red bars mark reported characteristic domains. Red arrow indicates a signal peptide cleavage site detected by SignalP 4.1 Server (Petersen et al. 2011). Suppl. Fig. S3 Reconstitution of ABA signaling pathway by co-transfection of AREB1, ABI1 and PYL1. Protoplasts were isolated from WT leaves. *RD29B*-*GUS* (5.0 μg of plasmid per transformation) and *pBI35SΩ*-*ELUC* (1.0 μg per transfection) were used as the ABA-responsive reporter and internal control, respectively. Each transfection used 2.5 μg of effector plasmid, except for ABI1, which used 1.5 μg per transfection. Total amounts of effector DNA were 6.5 μg, which include effector plasmids alone or combined with the vector control plasmid *pSKX* for transient expression analysis. ‘Relative activity’ indicates combined expression relative to the value obtained from the vector control. After transfection, protoplasts were incubated for 14-18 h under dark conditions without ABA (open bars) or with 2.0 μM ABA (filled bars). Error bars indicate SD (*n* = 4). Experiments were performed at least three times, and a representative result is shown (PDF 555 kb)

## Abbreviations

ABF: ABRE-binding factor
ABRE: ABA-responsive element
AREB: ABRE-binding protein
CK2: Casein kinase II
CK2s: Casein kinase II subunits
PP2C: Group-A protein phosphatase 2C
PYR1/PYL/RCAR: Pyrabactin resistance1/PYR1-like/regulatory components of ABA receptor
SnRK2: Subclass III sucrose nonfermenting 1 related protein kinase 2
