# Correction to: Casein kinase 2 α and β subunits inversely modulate ABA signal output in* Arabidopsis* protoplasts

**DOI:** 10.1007/s00425-018-3045-0

**Published:** 2018-11-17

**Authors:** Yukari Nagatoshi, Miki Fujita, Yasunari Fujita

**Affiliations:** 1 Biological Resources and Post-harvest Division, Japan International Research Center for Agricultural Sciences (JIRCAS), Tsukuba, Ibaraki 305-8686 Japan; 2 RIKEN Center for Sustainable Resource Science, Tsukuba, Ibaraki 305-0074 Japan; 3 Graduate School of Life and Environmental Sciences, University of Tsukuba, Tsukuba, Ibaraki 305-8572 Japan

## Correction to: Planta (2018) 248:571–578 10.1007/s00425-018-2919-5

The article Casein kinase 2 α and β subunits inversely modulate ABA signal output in *Arabidopsis* protoplasts, written by Yukari Nagatoshi, Miki Fujita, and Yasunari Fujita, was originally published electronically on the publisher’s internet portal (currently SpringerLink) on 24 May 2018 without open access. With the author(s)’ decision to opt for Open Choice the copyright of the article changed on 19 November 2018 to © The Author(s) 2018 and the article is forthwith distributed under the terms of the Creative Commons Attribution 4.0 International License (http://creativecommons.org/licenses/by/4.0/), which permits use, duplication, adaptation, distribution and reproduction in any medium or format, as long as you give appropriate credit to the original author(s) and the source, provide a link to the Creative Commons license and indicate if changes were made.

The original article has been corrected.

