# Supplementary material for: Casein kinase 2 α and β subunits inversely modulate ABA signal output in Arabidopsis protoplasts
Source: Planta. 2018 May 24;248(3):571–8. doi: 10.1007/s00425-018-2919-5 (PMC6244648; doi:10.1007/s00425-018-2919-5)
Supplement: Supplementary file 1 — Suppl. Table S1 Oligonucleotide primers used in this study. Suppl. Fig. S1 Phylogenetic tree of CK2αs and CK2βs in Arabidopsis. The neighbor-joining phylogenetic tree (Saitou and Nei 1987) was created using MEGA7 (Kumar et al. 2016). The optimal tree with the sum of branch length = 1.25324778 is shown. Bootstrap values (1000 replicates) are shown next to the branches (Felsenstein 1985). Evolutionary distances were computed using the p-distance method (Nei and Kumar 2000); units represent the number of amino acid differences per site. The subcellular localization of each CK2 is based on previous reports (Salinas et al. 2006; Perales et al. 2006; Portoles and Mas 2010: Mulekar and Huq 2015). CK2αs and CK2βs are shaded in pink and in yellow, respectively. Suppl. Fig. S2 Alignment of CK2 amino acid sequences in Arabidopsis. Comparison of amino acid sequences of CK2αs (a) and CK2βs (b). Conservation ratio at each site is shown by shading (black, 100%; gray, 75%). Red bars mark reported characteristic domains. Red arrow indicates a signal peptide cleavage site detected by SignalP 4.1 Server (Petersen et al. 2011). Suppl. Fig. S3 Reconstitution of ABA signaling pathway by co-transfection of AREB1, ABI1 and PYL1. Protoplasts were isolated from WT leaves. RD29B-GUS (5.0 μg of plasmid per transformation) and pBI35SΩ-ELUC (1.0 μg per transfection) were used as the ABA-responsive reporter and internal control, respectively. Each transfection used 2.5 μg of effector plasmid, except for ABI1, which used 1.5 μg per transfection. Total amounts of effector DNA were 6.5 μg, which include effector plasmids alone or combined with the vector control plasmid pSKX for transient expression analysis. ‘Relative activity’ indicates combined expression relative to the value obtained from the vector control. After transfection, protoplasts were incubated for 14-18 h under dark conditions without ABA (open bars) or with 2.0 μM ABA (filled bars). Error bars indicate SD (n = 4). Experiments were p [file 425_2018_2919_MOESM1_ESM.pdf]

Supplemental Table S1. Primers used in this study.

Primer Name	Seq (5'-3')
**Primers used to construct pSKX-based plasmids for transient expression analysis**	
pGKXEl2-5'PstHind	gtcctgcagaagcttGGAGCTTGCCAACA
pGrMCS-3'	GATGTGCTGCAAGGCGATTAAG
#5-CKA1.2s-BamHI	agaggatccATGATAGATACGCTTTTCTTCTTG
#12-CKA1.3as-SacI	tcgagctcTCATTGACTTCTCATTCTGCT
#7-CKA2.1s-BamHI	agaggatccATGCACCTAATCTTCTTCTTCTC
#13-CKA2.2as-SacI	tcgagctcCTATTGAGTCCTCATTCTGCTG
#1-CKA3.1s-BamHI	agaggatccATGTCGAAAGCTAGGGTTTA
#14-CKA3.2as-SacI	tcgagctcTTACTGAGTTCGTAGTCTGCTGC
#3-CKA4.1s-BamHI	agaggatccATGGCCTTAAGGCCTTG
#11-CKA4.3as-SacI	tcgagctcTCACTGGCTGCGCGGCGTACGGCTGCTCTCTGCATT
#15-CKB1.1s-BamHI	gaggatccATGTATAGAGACAGAGGAAC
#72 CKB1.1-as-sacI	tcgagctcTCACGGTTTGTGTAATTTG
#17-CKB2.1s-BamHI	gaggatccATGTATAGGGAGAGAGGTATG
#18-CKB2.1as-SacI	tcgagctcTCACGGCTTGTGTAG
#19-CKB3.1s-BamHI	gaggatccATGTACAAGGAACGTAG
#20-CKB3.1as-SacI	tcgagctcTCATGGTTTGTGTAC
#21-CKB4.1s-BamHI	gaggatccATGTACAAGGATCGG
#22-CKB4.1s-SacI	tcgagctcTCATTGTTTGTTGTGTAC
15ABI1F-NotI	cccgcggccgcATGGAGGAAGTATCTCCGGCGATC
16ABI1R1-StuI	CAGATCCTCCTCAGAAATCAGCTTTTGCTC*AGGCCT*GTTCAAGGGTTTGCTCTTGAG
31ABI1R4-dMyc-SmaI	CTTCTTCGCTTATTAATTTCTGTTCCAGATCCTCCTCAG
32ABI1R5-dMyc-SmaI	gcgcccgggTCATAAGTCTTCTTCGCTTATTAATTTCTG
27SRK2DF-NotIa	cccgcggccgcATGGATCCGGCGACTAATTC
28SRK2DR1-StuIa	GTCCTTGTAGTCAGAAGGCCTGAGAGCATAAACTATCTCTCC
14SRK2DR2-Flag-EcoRV	cccgatatcTCACTTGTCATCGTCGTCCTTGTAGTCAGAAG
29His-NcoI-PYL1F1a	CACCATCACccatggATGGCGAATTCAGAG
30PYL1R-EcoRVa	cccgatatcTTACCTAACCTGAGAAGAG
25NotI-His-NcoI-PYL1F2	cccgcggccgcATGAAACATCACCATCACCATCACccatggATG

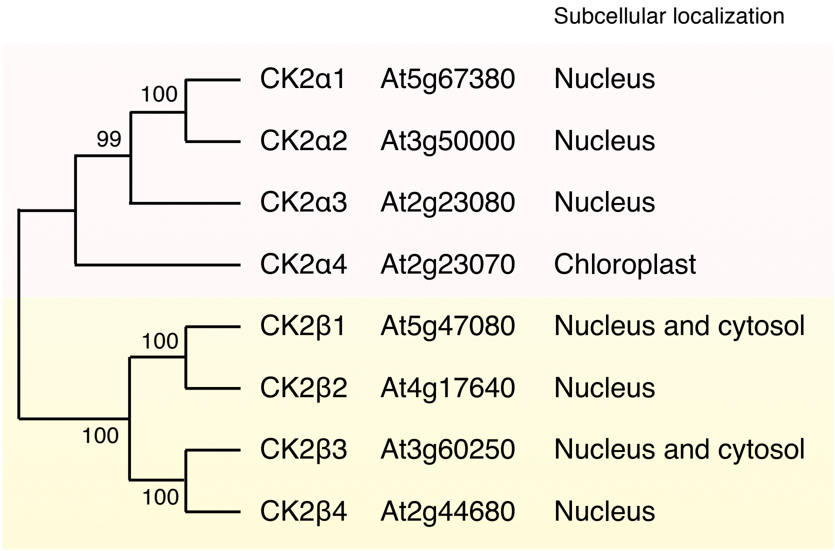

Supplemental Figure S1. Phylogenetic Tree of CK2αs and CK2βs in *Arabidopsis*
Phylogenetic Tree of CK2αs and CK2βs in *Arabidopsis.* The neighbor-joining phylogenetic tree (Saitou, Nei 1987) was created using MEGA7 (Kumar et al. 2016). The optimal tree with the sum of branch length = 1.25324778 is shown. Bootstrap values (1,000 replicates) are shown next to the branches (Felsenstein. 1985). Evolutionary distances were computed using the p-distance method (Nei, Kumar 2000); units represent the number of amino acid differences per site. The subcellular localization of each CK2 is based on previous reports (Salinas et al. 2006; Perales et al. 2006; Portoles, Mas 2010: Mulekar, Huq 2015). CK2αs and CK2βs are shaded in pink and in yellow, respectively.

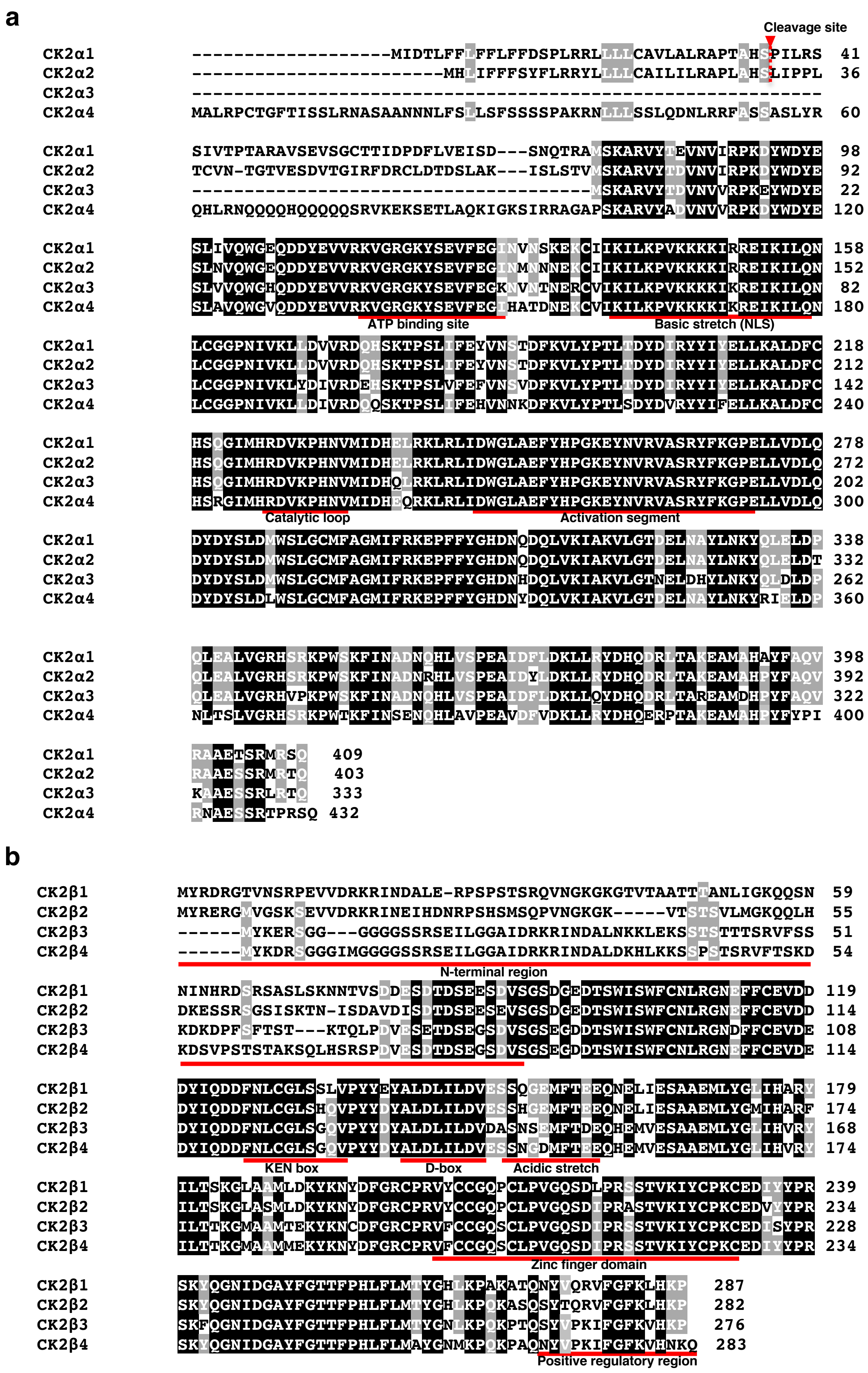

Supplemental Figure S2. Alignment of CK2 Amino Acid Sequences in *Arabidopsis*

Comparison of amino acid sequences of CK2αs **a** and CK2βs **b**. Conservation ratio at each site is shown by shading (black, 100%; gray, 75%). Red bars mark reported characteristic domains. Red arrow indicates a signal peptide cleavage site detected by SignalP 4.1 Server (Petersen et al. 2011).

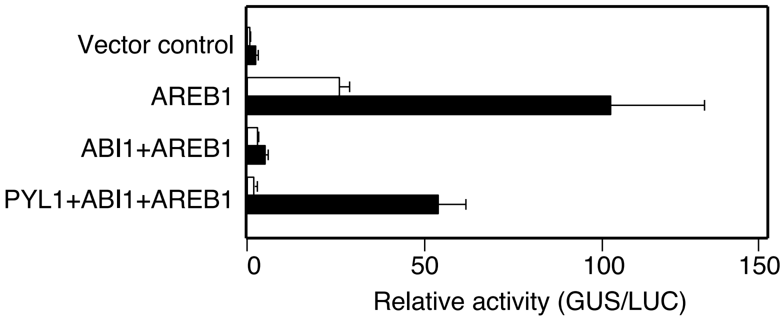

Supplemental Figure S3. Reconstitution of ABA signaling pathway by co-transfection of AREB1, ABI1 and PYL1.

Protoplasts were isolated from WT leaves. *RD29B-GUS* (5.0 µg of plasmid per transformation) and *pBI35SΩ-ELUC* (1.0 µg per transfection) were used as the ABA-responsive reporter and internal control, respectively. Each transfection used 2.5 µg of effector plasmid, except for ABI1, which used 1.5 µg per transfection. Total amounts of effector DNA were 6.5 µg, which include effector plasmids alone or combined with the vector control plasmid *pSKX* for transient expression analysis. 'Relative activity' indicates combined expression relative to the value obtained from the vector control. After transfection, protoplasts were incubated for 14-18 h under dark conditions without ABA (open bars) or with 2.0 µM ABA (filled bars). Error bars indicate SD (*n* = 4). Experiments were performed at least three times, and a representative result is shown.
